# Cerebrolith in hydranencephaly

**DOI:** 10.1007/s00247-025-06200-x

**Published:** 2025-02-25

**Authors:** Mehmet Atalar

**Affiliations:** Sivas Cumhuriyet University Faculty of Medicine, Department of Radiology, Sivas, Turkey

A 34-year-old woman, gravida 2, parity 1, was referred from an external center at 26 weeks’ gestation with a preliminary diagnosis of hydrocephalus. The patient underwent fetal magnetic resonance imaging (MRI). Coronal T2-weighted (T2W) half-Fourier acquisition single-shot turbo spin-echo (HASTE) MR image (**a**) shows hydranencephaly (*asterisk*). Posterior fossa structures are intact (*white arrow*). Remnants of occipital parenchymal structures are also visible (*black arrows*). Coronal T2W HASTE MR image (**b**) shows a cerebrolith as a hypointense nodular mass adjacent to the inner table of the occipital bone (*arrow*). The cerebrolith associated with hydranencephaly may represent infarcted brain tissue, which is consistent with the etiology of hydranencephaly.
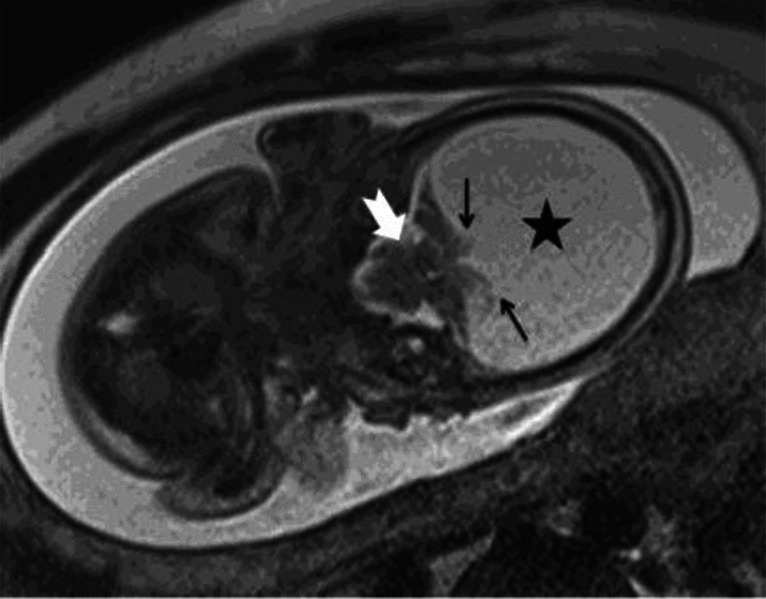



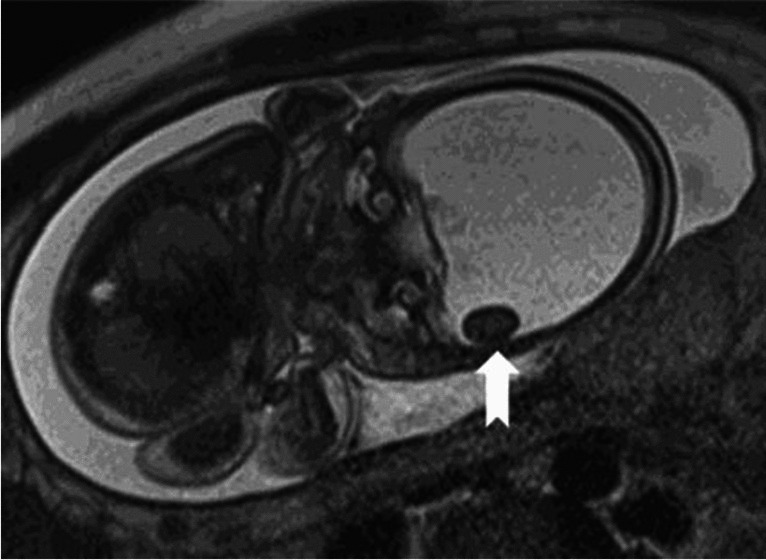


## Data Availability

No datasets were generated or analysed during the current study.

